# Molecular Integrative Clustering of Asian Gastric Cell Lines Revealed Two Distinct Chemosensitivity Clusters

**DOI:** 10.1371/journal.pone.0111146

**Published:** 2014-10-24

**Authors:** Meng Ling Choong, Shan Ho Tan, Tuan Zea Tan, Sravanthy Manesh, Anna Ngo, Jacklyn W. Y. Yong, Henry He Yang, May Ann Lee

**Affiliations:** 1 Experimental Therapeutics Centre, Agency for Science Technology and Research, Singapore, Singapore; 2 Bioinformatics Core, Cancer Science Institute of Singapore, National University of Singapore, Singapore; University of Nebraska Medical Center, United States of America

## Abstract

Cell lines recapitulate cancer heterogeneity without the presence of interfering tissue found in primary tumor. Their heterogeneous characteristics are reflected in their multiple genetic abnormalities and variable responsiveness to drug treatments. In order to understand the heterogeneity observed in Asian gastric cancers, we have performed array comparative genomic hybridization (aCGH) on 18 Asian gastric cell lines. Hierarchical clustering and single-sample Gene Set Enrichment Analysis were performed on the aCGH data together with public gene expression data of the same cell lines obtained from the Cancer Cell Line Encyclopedia. We found a large amount of genetic aberrations, with some cell lines having 13 fold more aberrations than others. Frequently mutated genes and cellular pathways are identified in these Asian gastric cell lines. The combined analyses of aCGH and expression data demonstrate correlation of gene copy number variations and expression profiles in human gastric cancer cells. The gastric cell lines can be grouped into 2 integrative clusters (ICs). Gastric cells in IC1 are enriched with gene associated with mitochondrial activities and oxidative phosphorylation while cells in IC2 are enriched with genes associated with cell signaling and transcription regulations. The two clusters of cell lines were shown to have distinct responsiveness towards several chemotherapeutics agents such as PI3 K and proteosome inhibitors. Our molecular integrative clustering provides insight into critical genes and pathways that may be responsible for the differences in survival in response to chemotherapy.

## Introduction

Gastric cancer is the second leading cause of cancer death worldwide, and is particularly common in East Asia [1]. It does not get as much attention as other cancers because of its lower incidence in the West. There is a decreasing trend in the incidence of this cancer. However, rates in Asia are among the highest in the world. It is the third most common cancer in males in Singapore and the fifth most common cancer in females in Singapore [2]. It claimed approximately 330 lives every year in Singapore. Diagnosis of gastric cancer usually occurs at late stage of the disease when treatment options are limited and often unsuccessful. Therefore it is critical to improve on early detection and treatment of the cancer.

Traditionally, classification of gastric cancers is based on histopathological findings. The widely used Lauren’s classification divides gastric cancers into two major histological types, namely intestinal type or diffuse type [3]. Intestinal type cancers have recognizable gland formation which ranges from well to poorly differentiated. The tumors grow in expanding, rather than infiltrative, patterns. They are believed to arise from chronic atrophic gastritis. In contrast, diffuse type cancers have noncohesive tumor cells diffusely infiltrating the stroma of the stomach and often exhibit deep infiltration of the stomach wall with little or no gland formation. They may arise out of single-cell mutations within normal gastric glands, and are associated with worse prognosis [4]. Advances in molecular biology have made available molecular classifications based either on genomic aberrations or gene expression profiles, or an integration of both [5]–[10].

Cell lines formed the foundation of cancer biology and the quest for drug treatments. Common alterations in cell lines, which include gains and losses of entire arms of chromosomes, are often the same ones found in primary tissue [11]. Cell lines also do not contain non-cancerous cells found in primary tumor tissues, making the cultivated lines ideal for finding mutations in the cancer genome [12]. Thus, comparing signatures between cell lines would more likely reflect intrinsic differences between tumor cells with minimal potentially confounding effects from neighboring non-cancer cells [5].

Many Asian gastric cell lines are available commercially and they have contributed to the progress in gastric cancer biology and treatment. Despite being relatively homogenous and devoid of tissue complexity, these Asian gastric cell lines are found to have heterogeneous response or susceptibility to drug treatment (personal unpublished data, and public data in CCLE and Sanger COSMIC). We reasoned that subtle genomic variations may contribute to the underlying differences observed among these gastric cell lines. Unlike tumor tissue, these differences would be intrinsic and a signature to the biology of the particular gastric cell line since there is no interference from other cell types.

We analyzed the gene copy number and LOH in 18 Asian gastric cell lines. Coupling our results to gene expression profiles of these cell lines available from CCLE, we identified two distinct genomic signatures based on genetic aberrations among these Asian gastric cancer cell lines. The clustering was further validated by in vitro chemotherapy sensitivity study done in our lab and with data publicly available from CCLE and Sanger COSMIC databases. A molecular classification on gastric cancer patients would have great clinical impact as it leads to more accurate prediction of prognosis, allowing targeted therapy based on the underlying biology of each subgroup.

## Materials and Methods

### Cell lines

Asian gastric cell lines SNU-1, SNU-5, SNU-16 and Kato-III, were purchased from the American Type Culture Collection. MKN1, MKN7, MKN45, MMKN74, Fu97, AZ-521, SCH, OCUM-1, NUGC-3, NUGC-4, IM95 and IM95 m were purchased from Japan Health Sciences Foundation. SNU-216 was purchased from Korea Cell Line Bank. YCC-3 [5] was a gift from Patrick Tan, Genome Institute of Singapore.

### Array comparative genomic hybridization (aCGH) and copy number determination

CGH array was performed by Origen Labs (Singapore) using the Affymetrix CytoScan HD array platform. Cell pellets containing 1×10^6^ cells were used for the aCGH hybridization. The genomic DNA quality, hybridization signal strengths and internal controls satisfied Affymetrix required standard at each step before proceeding to the next. Data from the 18 gastric cell lines on Affymetrix Cytoscan HD array were pre-processed using Affymetrix Chromosome Suite 1.2.2.271 using the single sample analysis workflow and default settings. Hidden Markov model segmentation was applied to call DNA copy number gain, loss or loss of heterozygosity (LOH) status. For matching purposes, the Entrez Gene ID was assigned to each gene name in this data set using the NCBI’s Entrez Gene Database [13]. DNA copy number gain, loss or LOH was compiled by counting the number of mutation for each cell line and mutation type. The kinome gene list and human pathways were downloaded from public databases [14], [15].

### Public microarray data pre-processing

Affymetrix U133 Plus2 DNA microarray gene expressions of 27 gastric cancer cell lines (Kato-III, IM95, SNU-620, SNU-16, OCUM-1, NUGC-4, 2313287, HUG1N, MKN45, NCIN87, KE39, AGS, SNU-5, SNU-216, NUGC-3, NUGC-2, MKN74, MKN7, RERFGC1B, GCIY, KE97, Fu97, SH10TC, MKN1, SNU-1, Hs746 T, HGC27) were downloaded from Cancer Cell Line Encyclopedia (CCLE) [16] in March 2013. Robust Multi-array Average (RMA) normalization was performed. Principal component analysis plot show no obvious batch effect. The normalized data is then collapsed by taking the probe sets with highest gene expression.

### Hierarchical clustering and single-sample Gene Set Enrichment Analysis (ssGSEA)

Unsupervised hierarchical clustering (1-Spearman distance, average linkage) was performed on the cell lines using the aCGH data. Putative driver genes of which copy number aberrations correlated to mRNA gene expression were identified to determine subtypes or clusters that are driven by different mechanisms. This was done using Mann Whitney U-test with p<0.05, and Spearman Correlation Coefficient test with Rho >0.6. We then performed consensus clustering [17] on the gene expression data of the 27 gastric cancer cell lines from CCLE using these putative driver genes. We selected k = 2 as it gives sufficiently stable similarity matrix. In order to assign new samples to this integrative cluster, significance analysis of microarray (SAM) [18] with threshold q<2.0 was used to generate subtype signature based on the mRNA expression data of the 1762 genes from the 27 gastric cancer cell lines in CCLE.

ssGSEA was used to estimate pathway activities of the gastric cancer cell line in the Molecular Signature Database v3.1 (Msigdb v3.1) [19], [20]. The pathway activities are represented in enrichment scores which were rank normalized to [0.0, 1.0]. SAM analysis was performed with threshold q<0.2, and fold change >2.0 (for up-regulated pathways), or <0.5 (for down-regulated pathways) to obtain subtype-specific pathways from the 27 gastric cell lines in CCLE.

### Drug treatment

A panel of 39 compounds against various cellular targets was obtained from Selleck Chemicals (Table S1 in File S1). Cells were seeded in 50 µl medium in 96-well plates at 8000 cells/well and incubated overnight. Serial dilutions of compounds were performed starting from 200 µM with 1: 4 dilutions for subsequent dilutions. Serially diluted compounds (50 µl) were added to cells and incubated for 48 hours. To measure cell viability, CellTiter-Glo (Promega) was added to the wells at 1∶1 ratio. After 10 minutes of incubation at room temperature, luminescence was measured with Safire II plate reader (Tecan). The experiments were carried out in triplicates for each dose dilution point and independently replicated on a separate occasion. Data was analyzed with Graphpad Prism software to determine the half maximal inhibitory concentration (IC_50_). *P*-values were computed from Mann Whitney *U*-test.

## Results and Discussions

### Genetic aberrations in the Asian gastric cell lines

In this study, we analyzed the copy number aberrations and LOH in Asian gastric cell lines by aCGH. Genes that are frequently gain, loss or have LOH are identified. Though continuous cell lines tend to harbor more mutations than the primary tumor where it was originally derived [6], cell lines are able to recapitulate major patterns of tumor heterogeneity [21], [22]. We detected total genetic aberrations (gain, loss and LOH) in the Asian gastric cell lines ranging from 1724 in AZ-521 to 22631 in NUGC-3 (Table 1). Reflecting this trend, we found that AZ-521 has the least number of genetic aberrations in the human kinome (37 out of a total of 531 kinases) [14] while NUGC-3 has the most number of gene aberrations (510) in its kinome (Table 1).

**Table 1:** Total and kinome genetics aberrations (consisting of gain, loss and LOH) in the 18 Asian gastric cell lines.

	Total Genetic Aberrations				Kinome Genetic Aberations			
Name	Gain	Loss	LOH	Total	Gain	Loss	LOH	Total
AZ-521	1	1582	141	1724	0	35	2	37
SNU-1	611	1718	76	2405	13	41	1	55
IM95	1819	2137	1155	5111	73	0	35	108
IM95 m	1828	2236	1161	5225	45	48	24	117
Kato-III	3135	0	2131	5266	45	51	24	120
SNU-5	3646	0	1983	5629	89	0	50	139
SNU-16	3195	2621	1130	6946	101	0	47	148
Fu97	4826	0	2319	7145	78	60	25	163
MKN45	2015	0	5339	7354	40	0	129	169
YCC-3	5767	0	2036	7803	134	0	37	171
NUGC-4	7151	0	664	7815	83	0	94	177
MKN7	3859	0	4047	7906	165	0	22	187
OCUM-1	6045	4416	57	10518	143	116	3	262
SCH	3048	8130	1803	12981	71	195	49	315
MKN1	4687	7885	2808	15380	115	174	64	353
MKN74	5733	9792	1935	17460	139	238	47	424
SNU-216	3574	12481	1834	17889	82	307	40	429
NUGC-3	7202	15355	74	22631	154	354	2	510

We found that 72% of the Asian gastric cell lines have gene copy number gain in *CDK13, EGFR, PAK1* and *STK17A* kinases (Table 2). Amplification of *CDK13* was found to associate with gastric and liver cancers [23]. High *EGFR* and *PAK1* expression levels are closely correlated to the incidence and development of gastric cancer in East Asians [24], [25] while an unanticipated role for STK17A as a candidate promoter of cell proliferation and survival was recently identified [26]. On the other hand, 44% of these cell lines have gene copy number loss in *MAP3K15*, an apoptosis-facilitating factor [27], and *RPS6KA6*, a potent tumor suppressor in multiple cancers [28]. LOH is associated with inactivation or loss of a normal allele. We detected LOH of *GUCY2F* in 61% of the Asian gastric cancer cell lines. *GUCY2F* is needed to repress transcription of several growth factor genes and inhibits growth of gastric carcinoma [29]. LOH of *MYLK3* is found in 56% of the Asian gastric cell lines. *MYLK3* is implicated in gastric acid secretion (KEGG entry 91807) and reduced secretion of gastric acid due to atrophic mucosa is observed in gastric cancer.

**Table 2:** Frequently mutated kinase genes in the 18 Asian gastric cell lines.

Gain		Loss		LOH	
Kinase	Number ofcell lines	Kinase	Number ofcell lines	Kinase	Number ofcell lines
*CDK13*	13 (72%)	*MAP3K15*	8 (44%)	*GUCY2F*	11 (61%)
*EGFR*	13	*RPS6KA6*	8	*PAK3*	11
*STK17A*	13	*PRKX*	8	*CDKL5*	11
*PHKG1*	12	*GUCY2F*	8	*MYLK3*	10
*CAMK2B*	12	*RPS6KA3*	8	*RPS6KA3*	10
*STK31*	12	*MST4*	8	*RPS6KA6*	10
*HCK*	11	*NRK*	8	*MAP3K15*	10
*SRC*	11	*BMX*	7	*WNK3*	10
*AURKA*	11	*TAF1*	7	*PIM2*	9
*ADCK5*	10	*PDK3*	7	*BTK*	9
*STK3*	10	*JAK2*	7	*CASK*	9
*SGK2*	10	*CASK*	7	*CDK16*	9
*PAK1*	10	*CDK16*	6	*PDK3*	9
*SRMS*	10	*ALPK2*	6	*ARAF*	9
*EIF2AK1*	10	*PAK3*	6	*PRKX*	9

**Figure 1:**
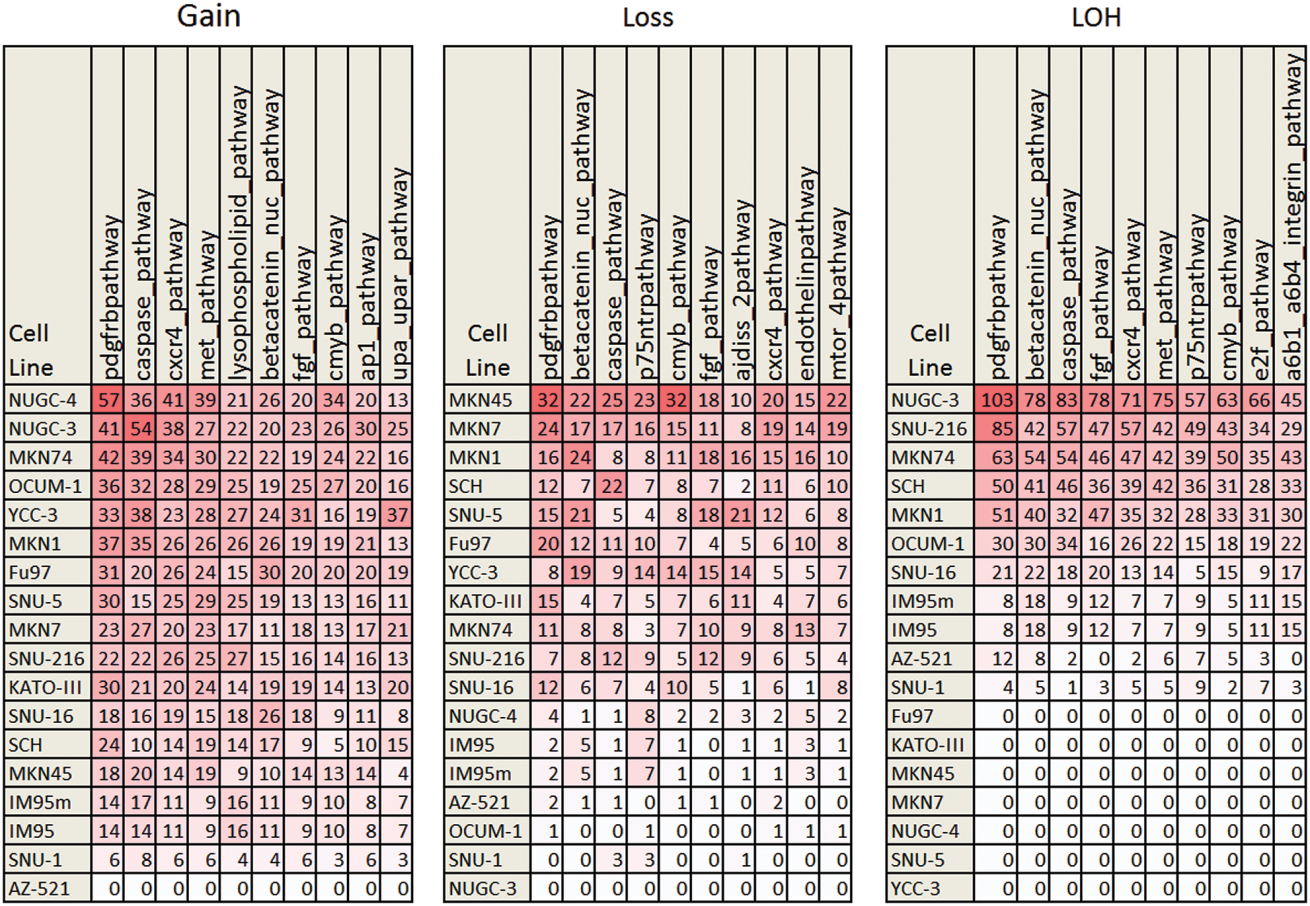
Top 10 cellular pathways having the most genetic aberrations in the Asian gastric cell lines. The numerics in red boxes are the number of aberrations. Increased intensity of red corresponds to increased number of aberrations.

The NCI-Nature Pathway Interaction Database [15] has 137 human pathways representing 9248 interactions. The top 10 pathways with the most number of gain, loss and LOH for each cell line from our array CGH analysis are summarized in Fig. 1. We found that genes in the PDGFR-beta and the caspase pathways contained the most number of genetic aberrations (gain, loss and LOH). Deregulation of the PDGFR-beta pathway affects angiogenesis in gastric cancer and depth of cancer cell invasion into the gastric subserosal layer [30]. Down-regulation of caspase activities has been detected in various human gastric cancer-derived cell lines [31]. On the other hand, genes in the nuclear beta-catenin pathway have the most number of loss and LOH with no gain of genetic materials in these Asian cell lines. Deregulation of the Wnt/beta-catenin pathway due to loss of membranous E-cadherin has been reported in gastric cancers [32].

### Integrative cluster identification and signature generation

**Figure 2:**
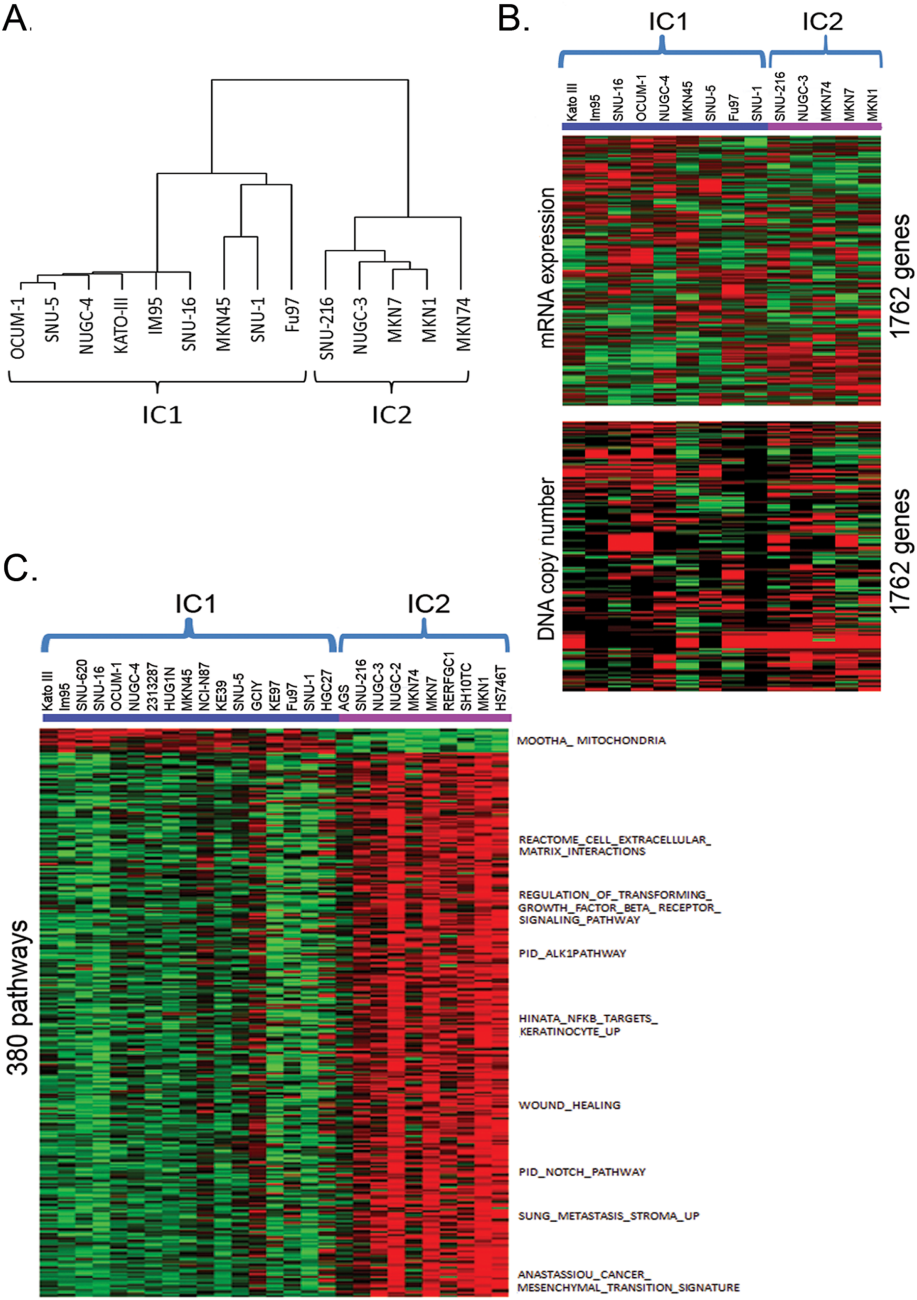
Molecular clustering of the Asian gastric cancer cell lines. (A) Hierarchical clustering using cell lines with both DNA copy number and mRNA expression data. (B) mRNA expression (mean-centered, normalized) heatmap (upper panel) and copy number (lower panel) of 1,762 putative driver genes from 14 gastric cell lines. (C) ssGSEA pathway enrichment score (mean-centered) heatmap for 380 subtype-specific pathways using 27 gastric cell lines from CCLE. Only selected pathway/genesets are labeled. Color code for mRNA expression: red = high expression, green = low expression. Color code for copy number: green = copy number loss, red = copy number gain, black = normal copy number. Color code for pathway enrichment: red = high enrichment, green = low enrichment.

Additionally, we investigated the correlation between copy number aberrations and gene expression data of these cell lines in the public domain. The integrated analysis of DNA copy number variations and corresponding gene expression data would allow identification of significant genes and cellular pathways critical to the gastric cancer pathophysiology. Of the 18 gastric cell lines that we have performed aCGH, only 14 cell lines have corresponding mRNA expression data in CCLE. CCLE has a total of 27 Asian gastric cell lines at the time of analysis (March 2013). We used Mann Whitney U-test and Spearman Correlation Coefficient test to identify 1762 putative driver genes of which copy number aberrations correlate to mRNA gene expression (Table S2 in File S1). Consensus clustering using these putative driver genes revealed 2 clusters of gastric cell lines. We named them integrated clusters (IC) 1 and 2 (Fig. 2A).

An overall strong correlation between DNA copy number and mRNA expression was observed (Fig. 2B). A similar study with human gastric tumor samples [6] also noted the correlation. These findings suggest that DNA copy number variation is a key contributor to the expression variation of these genes. Cells in IC1 have higher expression of genes involved in oxidoreductase and mitochondria activities. Cells in IC2 have higher expression of genes involved in diverse cellular signaling functions. The roles of these genes in the two clusters of gastric cancer would need to be explored further. SAM was then performed to generate 114 subtype signature genes based on the mRNA expression data of the 1762 genes from the 27 gastric cancer cell lines in CCLE (Table S3 in File S1).

The cell lines in our two integrative clusters correlated strongly with a molecular clustering system reported by Tan et al. [5]. Cell lines in our IC1 and IC2 groups are almost identical to cell lines in their G-INT and G-DIF groups, respectively. The only differences are cell lines Fu-97 and SNU-1 in our IC1 are grouped into G-DIF instead of G-INT. Tan et al. performed the classification based on gene expression data only while we incorporated both in-house aCGH data with public gene expression data. The additional genomic information from aCGH may result in re-arrangement of the hierarchical tree. Tan et al. also found that their data associated significantly with Lauren’s classification but remained distinct with overall concordance of 64% with Lauren’s histopathological classification. The discrepancies between molecular and histological classification could be due to the ability of genetic classification to capture salient features of the tumor that are less likely to be discerned by light microscopy [5].

### Pathway analysis for the Integrative Clusters

ssGSEA was used to estimate pathway activities of the gastric cancer cell line in the Msigdb v3.1. SAM analysis revealed 380 subtype-specific pathways (Table S4 in File S1). The pathway enrichment score heatmap of the 380 subtype-specific pathways from the 27 gastric cell lines in CCLE is shown in Fig. 2C. Cell lines in the IC1 cluster have enrichment of genes associated with oxidative phosphorylation and mitochondria functions. On the other hand, cell lines in the IC2 cluster have enrichment of genes associated with higher inflammatory response, epithelial-mesenchymal transition, TGF-beta, Notch, RAS, and NFκB signaling. Clustering of gastric cancers to IC1 emphasizes on metabolism and energy generation while IC2 emphasizes on cell signaling and regulation of transcriptions suggesting that there are two mechanistically very distinct groups of gastric cancers.

### Drug sensitivity of Asian gastric cell lines based on the integrative clustering

**Figure 3:**
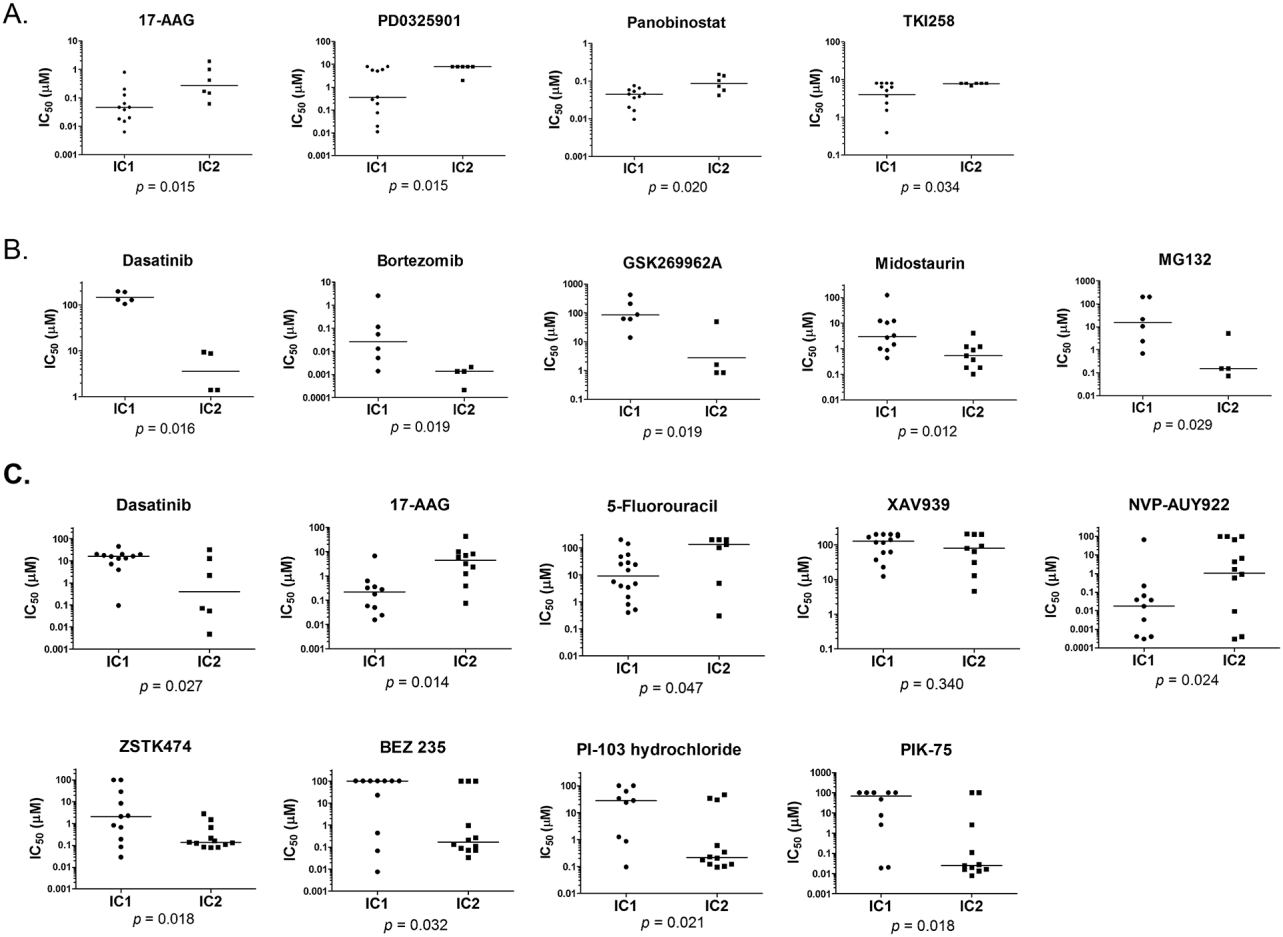
Dot plots of IC_50_ values for targeted inhibitors that have significant differences in toxicity to the Asian gastric cancer cells between the two integrated clusters. (A) Targeted inhibitors from the CCLE database. (B) Targeted inhibitors from the Sanger COSMIC database. (C) Selected targeted inhibitors in our lab showing significant differences in sensitivity (except XAV939) towards the two clusters of cell lines. Y-axis is the IC_50_ values in log10 scale. P-value is computed by Mann Whitney U-test. Horizontal bars are medians for sample distributions.

Drug sensitivity data (50% growth inhibitory concentration, IC_50_) were obtained from CCLE (Fig. 3A) and Sanger COSMIC (Fig. 3B). In both CCLE and COSMIC [33], cells were treated with compounds for approximately 72 hours. In-house drug sensitivity assay was performed for 48 hours (Table S5 in File S1). Growth inhibition between the two clusters of Asian gastric cancer cell lines was compared. Only compounds showing significant differential sensitivity between the two molecular clusters in CCLE, COSMIC and our in-house data are shown. We verified that the effect of 17-AAG and dasatinib in our collection of cell lines are similar to the results obtained from CCLE and COSMIC even though the length of incubation time with the compounds were different between our data and the public data (Fig. 3C).

5-Fluorouracil, a thymidylate synthase inhibitor which is the current treatment for gastric cancer, was found to be slightly more effective in cell lines in IC1 compared to IC2 (p = 0.047) (Fig. 3C). Similar results were also observed by Tan et al. [5] where cells in G-INT are more sensitive towards 5-fluorouracil than G-DIF. A significant benefit from adjuvant 5′-fluorouracil therapy in G-INT subtype compared to G-DIF subtype in retrospective patient cohorts has also been reported by Tan et al. Furthermore, a 10-year follow-up study found that 5-fluorouracil therapy with radiation could benefit all but the diffuse subtype based on Lauren’s classification [34].

Since we observed gene loss and LOH in the nuclear beta-catenin pathway, we postulate that targeting this pathway may have a therapeutic effect in gastric cancer [35]. However, we found that gastric cell lines in both IC1 and IC2 clusters are generally not responsive (IC_50_ ∼100 µM) towards XAV939, a tankyrase inhibitor [36] which selectively inhibit beta-catenin mediated transcription (Fig. 3C). This suggests that genetic aberrations in the beta-catenin pathway may be superfluous to the survival of the gastric cancer cells.

Cells in IC1 have enrichment of genes associated with oxidative phosphorylation and mitochondria functions. We found that cells in IC1 are more resistant to proteosome inhibitors bortezomib and MG132 (Fig. 3B). Proteosome inhibitors induce reactive oxygen species generation [37] which contribute to oxidative damage and cell death. Enrichment of genes associated with mitochondria function in cells in IC1 may enhance the ability of these cells to withstand oxidative damage. In contrast, the Hsp90 inhibitors, NVP-AUY922 and 17-AAG, are found to be more effective in inhibiting growth of cell lines in IC1 (p = 0.024 and 0.014, respectively). Mitochondrial Hsp90 is involved in complex signaling pathway that prevents initiation of induced apoptosis. The increased sensitivity of cells in the IC1 towards Hsp90 inhibitors further suggests that mitochondria activity is important in the survival of this cluster of cell lines [38].

Interestingly, we found that a subset of gastric cells within the IC1 are more sensitive towards the MEK/ERK inhibitor PD0325901 (p = 0.015; Fig. 3A). The MEK-ERK pathway is required for the S727 phosphorylation of mitochondrial STAT3 which is critical for electron transport chain activity and ATP abundance [39]. The pan histone deacetylase inhibitor panobinostat is also more toxic to gastric cells in IC1 (p = 0.020). On top of its mitochondrial modulatory effect and induction of apoptosis, panobinostat could also undermine the chaperon function of Hsp90 through hyperacetylation of Hsp90 [40]. Gastric cells in IC1 are also more sensitive towards TKI258 compared to cells in IC2 (p = 0.034). TKI258 is a multi-targeted receptor tyrosine kinase inhibitor with activity against FGFR, VEGFR, PDGFR, FLT3, and KIT. These will indirectly decrease Y243 phosphorylation of mitochondrial pyruvate dehydrogenase kinase 1, leading to inactivation of pyruvate dehydrogenase complex and decreased cell proliferation [41].

In support of our findings that the gastric cells in IC2 are enriched for genes involved in cell signalling, we found that cells in IC2 are generally more sensitive to kinase inhibitors than cells in IC1. Cell lines in IC2 are more sensitive to treatment with PI3 K inhibitors BEZ235, ZSTK424, PI-103 and PIK-75 (p = 0.032, 0.018, 0.021 and 0.018 respectively) (Figure 3C). This reflects a central role for the PI3 K pathway in cancer cell proliferation [42]. Targeting the PI3 K/AKT pathway may represent an important therapeutic target for gastric cancer [43]. We also found significantly lower IC_50_ values with gastric cells in IC2 compared to cells in IC1 when treated with kinase inhibitors dasatinib (Bcr-abl and Src inhibitor) (p = 0.027), GSK269962A (Rho kinase inhibitor) (p = 0.019), and midostaurin (Flt3 and multiple kinase inhibitor) (p = 0.012).

The absolute magnitude of the differential drug sensitivities ranges from 2–10 fold in the gastric cell lines based on our clustering. The modest differences may still be clinically meaningful given the small therapeutic windows associated with cytotoxicity even in targeted chemotherapy. A large patient cohort study will be needed to confirm the value of the molecular clustering strategies by us or others in predicting chemosensitivity and prognosis.

In conclusion, combination of aCGH and gene expression analysis to identify potential candidate oncogenes or tumor suppressor genes is a powerful and proven approach that has been reported in other cancer studies. This study provides insight into DNA copy number variations and their correlation to gene expression profiles in Asian gastric cell lines. A schematic diagram of the overall workflow is shown in Fig. S1 located in File S1. We report here the discovery of signature genes and cellular pathways associated with two genomic clusters of these cell lines. The two clusters of cell lines responded differentially to targeted therapeutic agents. Our results provide new insights into the molecular pathogenesis of this malignancy and could potentially augment the conventional histological classification of gastric cancers.

## Supporting Information

File S1 Table S1: A panel of target-specific compounds for chemosensitivity study in the Asian gastric cell lines. Table S2: Putative driver genes selected using correlated genes between copy number aberration and mRNA gene expression (Mann Whitney test, p<0.05, Spearman correlation coefficient, Rho >0.6). Table S3: Integrative cluster signature (Copy number and mRNA correlated genes). Table S4: Integrative cluster-specific pathway. Table S5: Compounds showing significant differences in sensitivity between the two integrative clusters of Asian gastric cell lines. Figure S1: A schematic diagram of the analysis workflow.
(XLSX)

## References

[1] Leung WK, Wu MS, Kakugawa Y, Kim JJ, Yeoh KG, et al (2008) Screening for gastric cancer in Asia: current evidence and practice. Lancet Oncol 9: 279–287. 18308253 10.1016/S1470-2045(08)70072-X

[2] Singapore Cancer Registry (2011) Cancer Survival in Singapore 1968–2007. Singapore: National Registry of Diseases Office: Health Promotion Board.

[3] Lauren P (1965) The Two Histological Main Types of Gastric Carcinoma: Diffuse and So-Called Intestinal-Type Carcinoma. An Attempt at a Histo-Clinical Classification. Acta Pathol Microbiol Scand 64: 31–49. 14320675 10.1111/apm.1965.64.1.31

[4] Dicken BJ, Bigam DL, Cass C, Mackey JR, Joy AA, et al (2005) Gastric adenocarcinoma: review and considerations for future directions. Ann Surg 241: 27–39. 15621988 10.1097/01.sla.0000149300.28588.23 PMC1356843

[5] Tan IB, Ivanova T, Lim KH, Ong CW, Deng N, et al. (2011) Intrinsic subtypes of gastric cancer, based on gene expression pattern, predict survival and respond differently to chemotherapy. Gastroenterology 141: 476–485, 485 e471–411. 10.1053/j.gastro.2011.04.042 PMC3152688 21684283

[6] Fan B, Dachrut S, Coral H, Yuen ST, Chu KM, et al (2012) Integration of DNA copy number alterations and transcriptional expression analysis in human gastric cancer. PLoS One 7: e29824. 22539939 10.1371/journal.pone.0029824 PMC3335165

[7] Tay ST, Leong SH, Yu K, Aggarwal A, Tan SY, et al (2003) A combined comparative genomic hybridization and expression microarray analysis of gastric cancer reveals novel molecular subtypes. Cancer Res 63: 3309–3316. 12810664

[8] Kim B, Bang S, Lee S, Kim S, Jung Y, et al (2003) Expression profiling and subtype-specific expression of stomach cancer. Cancer Res 63: 8248–8255. 14678982

[9] Chen X, Leung SY, Yuen ST, Chu KM, Ji J, et al (2003) Variation in gene expression patterns in human gastric cancers. Mol Biol Cell 14: 3208–3215. 12925757 10.1091/mbc.E02-12-0833 PMC181561

[10] Boussioutas A, Li H, Liu J, Waring P, Lade S, et al (2003) Distinctive patterns of gene expression in premalignant gastric mucosa and gastric cancer. Cancer Res 63: 2569–2577. 12750281

[11] Beroukhim R, Mermel CH, Porter D, Wei G, Raychaudhuri S, et al (2010) The landscape of somatic copy-number alteration across human cancers. Nature 463: 899–905. 20164920 10.1038/nature08822 PMC2826709

[12] Bignell GR, Greenman CD, Davies H, Butler AP, Edkins S, et al (2010) Signatures of mutation and selection in the cancer genome. Nature 463: 893–898. 20164919 10.1038/nature08768 PMC3145113

[13] Coordinators NR (2014) Database resources of the National Center for Biotechnology Information. Nucleic Acids Res 42: D7–17. 24259429 10.1093/nar/gkt1146 PMC3965057

[14] Manning G, Whyte DB, Martinez R, Hunter T, Sudarsanam S (2002) The protein kinase complement of the human genome. Science 298: 1912–1934. 12471243 10.1126/science.1075762

[15] Schaefer CF, Anthony K, Krupa S, Buchoff J, Day M, et al (2009) PID: the Pathway Interaction Database. Nucleic Acids Res 37: D674–679. 18832364 10.1093/nar/gkn653 PMC2686461

[16] Barretina J, Caponigro G, Stransky N, Venkatesan K, Margolin AA, et al (2012) The Cancer Cell Line Encyclopedia enables predictive modelling of anticancer drug sensitivity. Nature 483: 603–607. 22460905 10.1038/nature11003 PMC3320027

[17] Wilkerson MD, Hayes DN (2010) ConsensusClusterPlus: a class discovery tool with confidence assessments and item tracking. Bioinformatics 26: 1572–1573. 20427518 10.1093/bioinformatics/btq170 PMC2881355

[18] Tusher VG, Tibshirani R, Chu G (2001) Significance analysis of microarrays applied to the ionizing radiation response. Proc Natl Acad Sci U S A 98: 5116–5121. 11309499 10.1073/pnas.091062498 PMC33173

[19] Subramanian A, Tamayo P, Mootha VK, Mukherjee S, Ebert BL, et al (2005) Gene set enrichment analysis: a knowledge-based approach for interpreting genome-wide expression profiles. Proc Natl Acad Sci U S A 102: 15545–15550. 16199517 10.1073/pnas.0506580102 PMC1239896

[20] Verhaak RG, Tamayo P, Yang JY, Hubbard D, Zhang H, et al (2013) Prognostically relevant gene signatures of high-grade serous ovarian carcinoma. J Clin Invest 123: 517–525. 23257362 10.1172/JCI65833 PMC3533304

[21] Neve RM, Chin K, Fridlyand J, Yeh J, Baehner FL, et al (2006) A collection of breast cancer cell lines for the study of functionally distinct cancer subtypes. Cancer Cell 10: 515–527. 17157791 10.1016/j.ccr.2006.10.008 PMC2730521

[22] Hoshida Y, Toffanin S, Lachenmayer A, Villanueva A, Minguez B, et al (2010) Molecular classification and novel targets in hepatocellular carcinoma: recent advancements. Semin Liver Dis 30: 35–51. 20175032 10.1055/s-0030-1247131 PMC3668687

[23] Kim HE, Kim DG, Lee KJ, Son JG, Song MY, et al (2012) Frequent amplification of CENPF, GMNN and CDK13 genes in hepatocellular carcinomas. PLoS One 7: e43223. 22912832 10.1371/journal.pone.0043223 PMC3418236

[24] Gao M, Liang XJ, Zhang ZS, Ma W, Chang ZW, et al (2013) Relationship between expression of EGFR in gastric cancer tissue and clinicopathological features. Asian Pac J Trop Med 6: 260–264. 23608326 10.1016/S1995-7645(13)60054-1

[25] Li LH, Luo Q, Zheng MH, Pan C, Wu GY, et al (2012) P21-activated protein kinase 1 is overexpressed in gastric cancer and induces cancer metastasis. Oncol Rep 27: 1435–1442. 22293972 10.3892/or.2012.1664

[26] Mao P, Hever-Jardine MP, Rahme GJ, Yang E, Tam J, et al (2013) Serine/threonine kinase 17A is a novel candidate for therapeutic targeting in glioblastoma. PLoS One 8: e81803. 24312360 10.1371/journal.pone.0081803 PMC3842963

[27] Kaji T, Yoshida S, Kawai K, Fuchigami Y, Watanabe W, et al (2010) ASK3, a novel member of the apoptosis signal-regulating kinase family, is essential for stress-induced cell death in HeLa cells. Biochem Biophys Res Commun 395: 213–218. 20362554 10.1016/j.bbrc.2010.03.164

[28] Li Q, Jiang Y, Wei W, Ji Y, Gao H, et al (2014) Frequent epigenetic inactivation of RSK4 by promoter methylation in cancerous and non-cancerous tissues of breast cancer. Med Oncol 31: 793. 24338215 10.1007/s12032-013-0793-3

[29] Kitadai Y, Yamazaki H, Yasui W, Kyo E, Yokozaki H, et al (1993) GC factor represses transcription of several growth factor/receptor genes and causes growth inhibition of human gastric carcinoma cell lines. Cell Growth Differ 4: 291–296. 8494791

[30] Suzuki S, Dobashi Y, Hatakeyama Y, Tajiri R, Fujimura T, et al (2010) Clinicopathological significance of platelet-derived growth factor (PDGF)-B and vascular endothelial growth factor-A expression, PDGF receptor-beta phosphorylation, and microvessel density in gastric cancer. BMC Cancer 10: 659. 21118571 10.1186/1471-2407-10-659 PMC3009982

[31] Philchenkov A, Zavelevich M, Kroczak TJ, Los M (2004) Caspases and cancer: mechanisms of inactivation and new treatment modalities. Exp Oncol 26: 82–97. 15273659

[32] Cheng XX, Wang ZC, Chen XY, Sun Y, Kong QY, et al (2005) Frequent loss of membranous E-cadherin in gastric cancers: A cross-talk with Wnt in determining the fate of beta-catenin. Clin Exp Metastasis 22: 85–93. 16132582 10.1007/s10585-005-4578-8

[33] Garnett MJ, Edelman EJ, Heidorn SJ, Greenman CD, Dastur A, et al (2012) Systematic identification of genomic markers of drug sensitivity in cancer cells. Nature 483: 570–575. 22460902 10.1038/nature11005 PMC3349233

[34] Macdonald JS, Benedetti J, Smalley S, Haller D, Hundahl S, et al. (2009) Chemoradiation of resected gastric cancer: A 10-year follow-up of the phase III trial INT0116 (SWOG 9008). Journal of Clinical Oncology 27: abstr 4515.

[35] Wu WK, Cho CH, Lee CW, Fan D, Wu K, et al (2010) Dysregulation of cellular signaling in gastric cancer. Cancer Lett 295: 144–153. 20488613 10.1016/j.canlet.2010.04.025

[36] Huang SM, Mishina YM, Liu S, Cheung A, Stegmeier F, et al (2009) Tankyrase inhibition stabilizes axin and antagonizes Wnt signalling. Nature 461: 614–620. 19759537 10.1038/nature08356

[37] Ling YH, Liebes L, Zou Y, Perez-Soler R (2003) Reactive oxygen species generation and mitochondrial dysfunction in the apoptotic response to Bortezomib, a novel proteasome inhibitor, in human H460 non-small cell lung cancer cells. J Biol Chem 278: 33714–33723. 12821677 10.1074/jbc.M302559200

[38] Siegelin MD (2013) Inhibition of the mitochondrial Hsp90 chaperone network: a novel, efficient treatment strategy for cancer? Cancer Lett 333: 133–146. 23376257 10.1016/j.canlet.2013.01.045

[39] Gough DJ, Koetz L, Levy DE (2013) The MEK-ERK pathway is necessary for serine phosphorylation of mitochondrial STAT3 and Ras-mediated transformation. PLoS One 8: e83395. 24312439 10.1371/journal.pone.0083395 PMC3843736

[40] Rao R, Nalluri S, Fiskus W, Savoie A, Buckley KM, et al (2010) Role of CAAT/enhancer binding protein homologous protein in panobinostat-mediated potentiation of bortezomib-induced lethal endoplasmic reticulum stress in mantle cell lymphoma cells. Clin Cancer Res 16: 4742–4754. 20647473 10.1158/1078-0432.CCR-10-0529 PMC2948590

[41] Hitosugi T, Fan J, Chung TW, Lythgoe K, Wang X, et al (2011) Tyrosine phosphorylation of mitochondrial pyruvate dehydrogenase kinase 1 is important for cancer metabolism. Mol Cell 44: 864–877. 22195962 10.1016/j.molcel.2011.10.015 PMC3246218

[42] Porta C, Paglino C, Mosca A (2014) Targeting PI3 K/Akt/mTOR Signaling in Cancer. Front Oncol 4: 64. 24782981 10.3389/fonc.2014.00064 PMC3995050

[43] Ye B, Jiang LL, Xu HT, Zhou DW, Li ZS (2012) Expression of PI3 K/AKT pathway in gastric cancer and its blockade suppresses tumor growth and metastasis. Int J Immunopathol Pharmacol 25: 627–636. 23058013 10.1177/039463201202500309

